# Racemization of the substrate and product by serine palmitoyltransferase from *Sphingobacterium multivorum* yields two enantiomers of the product from d-serine

**DOI:** 10.1016/j.jbc.2024.105728

**Published:** 2024-02-05

**Authors:** Hiroko Ikushiro, Takumi Honda, Yuta Murai, Taiki Murakami, Aya Takahashi, Taiki Sawai, Haruna Goto, Shin-ichi Ikushiro, Ikuko Miyahara, Yoshio Hirabayashi, Nobuo Kamiya, Kenji Monde, Takato Yano

**Affiliations:** 1 Department of Biochemistry, Faculty of Medicine, Osaka Medical and Pharmaceutical University, Osaka, Japan; 2 Graduate School of Life Science, Hokkaido University, Sapporo, Japan; 3 Frontier Research Center for Advanced Material and Life Science, Faculty of Advanced Life Science, Hokkaido University, Sapporo, Hokkaido, Japan; 4 Division of Applied Bioscience, Graduate School of Agriculture, Hokkaido University, Sapporo, Hokkaido, Japan; 5 Department of Chemistry, Graduate School of Science, Osaka Metropolitan University, Osaka, Japan; 6 Department of Biotechnology, Faculty of Engineering, Toyama Prefectural University, Imizu, Toyama, Japan; 7 RIKEN Cluster for Pioneering Research, RIKEN, Wako, Saitama, Japan; 8 Institute for Environmental and Gender-Specific Medicine, Juntendo University Graduate School of Medicine, Chiba, Japan; 9 Research Center for Artificial Photosynthesis, Osaka Metropolitan University, Osaka, Japan

**Keywords:** serine palmitoyltransferase, sphingolipid, racemization, PLP-dependent enzyme, crystal structure, X-ray crystallography

## Abstract

Serine palmitoyltransferase (SPT) catalyzes the pyridoxal-5′-phosphate (PLP)-dependent decarboxylative condensation of l-serine and palmitoyl-CoA to form 3-ketodihydrosphingosine (KDS). Although SPT was shown to synthesize corresponding products from amino acids other than l-serine, it is still arguable whether SPT catalyzes the reaction with d-serine, which is a question of biological importance. Using high substrate and enzyme concentrations, KDS was detected after the incubation of SPT from *Sphingobacterium multivorum* with d-serine and palmitoyl-CoA. Furthermore, the KDS comprised equal amounts of *2S* and *2R* isomers. ^1^H-NMR study showed a slow hydrogen–deuterium exchange at Cα of serine mediated by SPT. We further confirmed that SPT catalyzed the racemization of serine. The rate of the KDS formation from d-serine was comparable to those for the α-hydrogen exchange and the racemization reaction. The structure of the d-serine–soaked crystal (1.65 Å resolution) showed a distinct electron density of the PLP–l-serine aldimine, interpreted as the racemized product trapped in the active site. The structure of the α-methyl-d-serine–soaked crystal (1.70 Å resolution) showed the PLP–α-methyl-d-serine aldimine, mimicking the d-serine–SPT complex prior to racemization. Based on these enzymological and structural analyses, the synthesis of KDS from d-serine was explained as the result of the slow racemization to l-serine, followed by the reaction with palmitoyl-CoA, and SPT would not catalyze the direct condensation between d-serine and palmitoyl-CoA. It was also shown that the *S. multivorum* SPT catalyzed the racemization of the product KDS, which would explain the presence of (*2R*)-KDS in the reaction products.

Serine palmitoyltransferase (SPT) is a key enzyme of sphingolipid biosynthesis and catalyzes the pyridoxal-5′-phosphate (PLP)-dependent decarboxylative condensation reaction between l-serine and palmitoyl-CoA to form 3-ketodihydrosphingosine (KDS), also called a long-chain base (LCB), as a common precursor of all sphingolipids (1, 2). Eukaryotic SPT functions as a membrane-bound large protein complex composed of SPTLC1/SPTLC2- or SPTLC1/SPTLC3-core dimer (3, 4, 5, 6, 7, 8, 9) and small regulatory subunits, ssSPTa or ssSPTb (10, 11, 12) and ORMD3 proteins (13, 14). Bacterial SPTs from sphingolipid-containing bacteria such as *Sphingomonas paucimobilis*, which is rich in glycosphingolipids, and *Sphingobacterium multivorum*, which is rich in phosphosphingolipids, function as a water-soluble homodimer enzyme and are useful as a prototypical model system of human SPT (15, 16). In addition to the high-resolution X-ray crystal structures of microbial SPTs (17, 18, 19, 20, 21, 22, 23), the structures of eukaryote SPT complexes were recently determined by cryo-EM at a resolution range of 2.6 to 3.8 Å (24, 25, 26). The active site architecture of the human SPT thus determined was indicated to be almost the same as that of the bacterial enzymes. Specifically, in SPTLC2 subunit of the human SPT, the ε-amino group of Lys379 forms a Schiff base with PLP, the carboxy group of Asp344 located at the bottom of the enzyme active site interacts with N1 of the PLP pyridine ring, and the side chain of His347 interacts with O3 of PLP by a hydrogen bond. The imidazole ring of His263 stacks to the PLP pyridine ring in parallel, and Ala346 forms a van der Waals interaction with the other side of the pyridine ring of PLP. All of these amino acid residues are fully conserved in the active site of bacterial SPTs (17, 22, 23).

Complete deficiency of SPT activity has not been reported because sphingolipids are arguably essential for cell survival. SPT activity is appropriately regulated to ensure homeostasis of sphingolipids to prevent cytotoxicity due to overproduction of certain sphingolipids. SPT, even the WT enzyme, can metabolize l-alanine and glycine in addition to its native substrate, l-serine, to form 1-deoxy type of LCB (23, 27, 28). Some mutations of the genes of SPT subunits are thought to shift its amino acid usage from l-serine to l-alanine, result in elevated levels of atypical 1-deoxysphingolipids, and cause the hereditary sensory and autonomic neuropathy type I (29, 30, 31). SPT overactivity due to another cluster of mutations causes a motor neuron disease, the childhood-onset amyotrophic lateral sclerosis (32). Elevated levels of 1-deoxysphingolipids have been reported to be associated with various metabolic syndromes such as macular disease, diabetes mellitus, cardiovascular disease, and tumor growth (33, 34, 35, 36, 37).

d-Serine, an optical isomer of l-serine, is known to act as an endogenous coagonist for synaptic *N*-methyl-d-aspartate type of glutamate receptor (38, 39). d-Serine is known to be synthesized in neurons by serine racemase, and its concentration in the cerebrum has been reported to be 0.27 μmol/g tissue, which is one third the concentration of l-serine (0.89 μmol/g tissue) (40). Disrupted homeostasis of serine enantiomers is suggested to be involved in the pathophysiology of neuropsychiatric disorders such as Alzheimer's disease (41), amyotrophic lateral sclerosis (42), schizophrenia (43), and depression (44) and is attracting attention as a candidate for a new diagnostic biomarker for these diseases.

Against this background, the relationship between d-serine and SPT, the first enzyme of the biosynthesis of all cellular sphingolipids, has been a persistent question among researchers: is d-serine really not metabolized by SPT? On the one hand, it has been reported that KDS formation from d-serine by SPT cannot be detected under the physiological reaction conditions and that d-serine acts only as a competitive inhibitor of mammalian SPT in the CHO cell culture system (1, 45). On the other hand, we previously reported that *S*. *multivorum* SPT could metabolize not only glycine and l-alanine but also l-homoserine, which is bulkier than l-serine (23), into the corresponding LCB products and form the external aldimine intermediate with a variety of amino acids or amine ligands, such as tris(hydroxymethyl)aminomethane, indicating a relatively promiscuous activity of this enzyme (22, 46). Here, we re-examined the reactivity of the *S. multivorum* SPT with d-serine to answer the above question and discussed the results based on the enzymatic and structural insights on SPT.

## Results

### Formation of KDS from d-serine by the *S. multivorum* SPT

**Figure 1:**
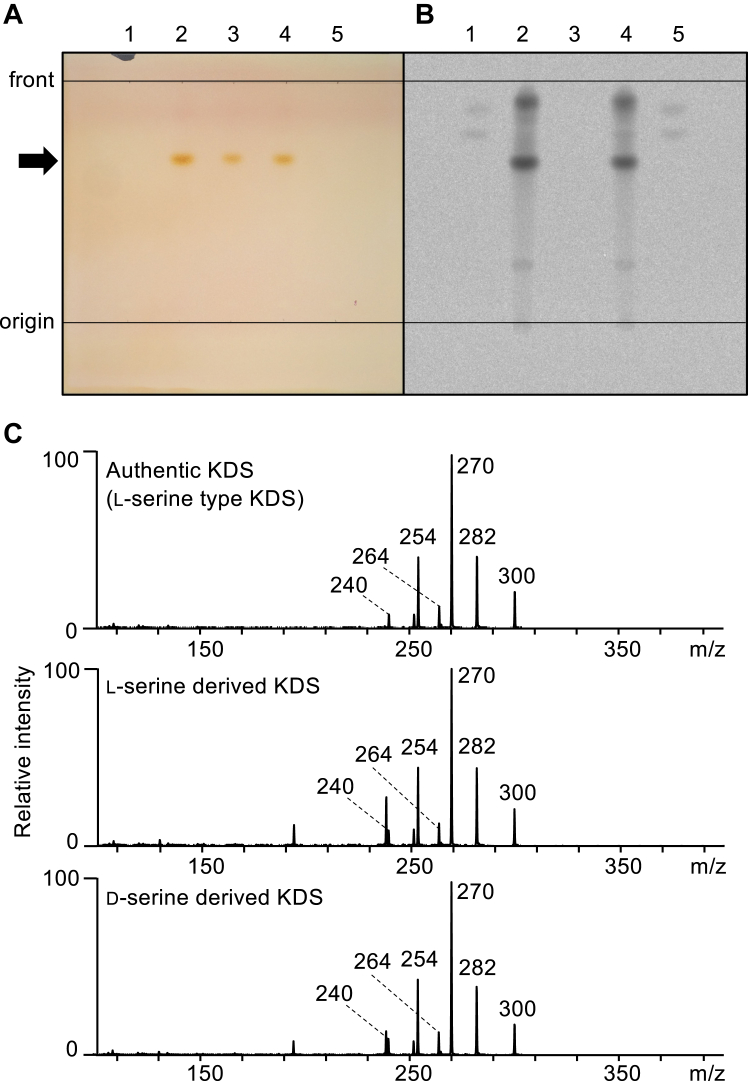
**Formation of KDS from l-serine and d-serine by the *Sphingobacterium multivorum* SPT.***A*, image of the TLC analysis of KDS visualized with ninhydrin reagent: *lane 1*, extract of the reaction mixture without the amino acid substrate; *lane 2*, extract of the reaction mixture with l-serine; *lane 3*, commercial authentic KDS (l-serine–derived, *(2S)*-KDS); *lane* 4, extract of the reaction mixture with d-serine; *lane 5*, extract of the reaction mixture without SPT. The complete reaction mixture contained 500 mM l-/d-serine, 1 mM palmitoyl-CoA, and 100 μM SPT and was incubated at 37 °C for 60 min. *B*, autoradiographic analysis of the same TLC plate. Radioactivity was incorporated to the reaction product from the substrate [1-^14^C]-palmitoyl-CoA. Each lane is the same as that in *panel A*. *C*, LC-MS/MS data for the reaction product. *Top*, commercial authentic KDS (l-serine–derived, *(2S)*-KDS); *middle*, l-serine–derived KDS; *bottom*, d-serine–derived KDS. KDS, 3-ketodihydrosphingosine; PLP, pyridoxal-5′-phosphate; SPT, serine palmitoyltransferase.

We previously reported that the KDS formation from d-serine was not detected under physiological assay conditions using *S*. *paucimobilis* SPT (15, 47). However, when 100 μM *S. multivorum* SPT was incubated with either 500 mM l-serine or d-serine in the presence of 1 mM [1-^14^C]-labeled palmitoyl-CoA, the reaction product was formed not only from l-serine but also from d-serine (Fig. 1). Each reaction product was ninhydrin-positive with the specific color and the Rf value, which corresponded to those of a commercial authentic KDS on the TLC plate (Fig. 1*A*, *lanes 2* and *4*). Both spots of the reaction products were highly radioactive, indicating that they carry the acyl group derived from [1-^14^C]-labeled palmitoyl-CoA (Fig. 1*B*). Such radioactive reaction products were not detected under the reaction condition lacking the amino acid substrate or SPT. The structure of the reaction product derived from each serine enantiomer was confirmed by HPLC/triple quadrupole/linear ion trap mass spectrometry using a commercially available authentic KDS as the standard (Fig. 1*C*). A positive ion MS/MS spectrum of the *m/z* 300 ion showed the presence of ions of *m/z* 282 and 270, resulting from the neutral loss of H_2_O and HCHO, respectively, which is consistent with a previous report (16).

Dependency of the reaction rates on amino acid concentrations under the condition of 1 mM palmitoyl-CoA was examined (Table 1). Each LCB product was quantified by the fluorescence intensity based on the standard curve obtained by using the corresponding authentic compound (23). The value of apparent catalytic efficiency ($k_{cat}^{app}/K_{m}^{app}$) for d-serine was 0. 0013 min^–1^ mM^–1^ and was much smaller than that for l-serine ($k_{cat}^{app}/K_{m}^{app}$ = 3.6 min^–1^ mM^–1^). Therefore, the KDS production by SPT from d-serine is considered to be negligible at the physiological concentration of d-serine. Next, binding of d-serine to the *S. multivorum* SPT was examined by spectroscopic titration. When SPT was incubated with d-serine, spectral changes corresponding to the external aldimine formation between PLP and the amino acid were observed (Supporting information). As shown in Table 1, the *K*_d_ value for d-serine was 11 ± 1.8 mM and was more than 20 times larger than that for l-serine (0.45 ± 0.050 mM).

**Table 1:** Apparent kinetic parameters of the *Sphingobacterium multivorum* SPT

Compounds	$k_{cat}^{app}$^a^[min^–1^]	$K_{m}^{app}$^a^[mM]	$k_{cat}^{app}/K_{m}^{app}$[min^–1^ mM^–1^]	$K_{d}$ [mM]
l-serine	13 ± 1.0^b^	3.6 ± 0.87^b^	3.6^b^	0.45 ± 0.050^b^
d-serine	0.31 ± 0.02	237.7 ± 44.3	0.0013	11 ± 1.8
α-methyl-l-serine	n.d.^c^	n.d.^c^	n.d.^c^	8.3 ± 1.8
α-methyl-d-serine	n.d.^c^	n.d.^c^	n.d.^c^	27 ± 2.5
glycine	0.008 ± 0.002^b^	77 ± 8.1^b^	0.0001^b^	71 ± 5.5^b^
l-alanine^d^	0.42 ± 0.011^b^	110 ± 9.0^b^	0.0037^b^	65 ± 10^b^
d-alanine	n.d.^c^	n.d.^c^	n.d.^c^	≫170^d^
l-homoserine	4.5 ± 0.28^b^	82 ± 14^b^	0.055^b^	3.6 ± 0.20^b^
d-homoserine	n.d.^c^	n.d.^c^	n.d.^c^	90 ± 8.0
l-threonine	n.d.^c^	n.d.^c^	n.d.^c^	8.2 ± 1.0^b^
Tris	n.d.^c^	n.d.^c^	n.d.^c^	40 ± 5.6^b^

Data are shown as mean ± SD of three or more measurements.

a The $k_{cat}^{app}$ and $K_{m}^{app}$ values were determined in the presence of 1 mM palmitoyl-CoA.

b Data from (21).

c n.d., not determined.

d No spectral changes were observed up to 170 mM d-alanine.

Because both l-homoserine and l-alanine could be converted to LCBs by SPT (22), the dissociation constants for enantiomers of these amino acids were also examined by the titration experiment. The *K*_d_ value for d-homoserine was 90 ± 8 mM, and no significant spectral changes were observed up to 170 mM of d-alanine. Each *K*_d_ value for the d-amino acid was much larger than that for the corresponding l-amino acid, l-homoserine (3.6 ± 0.20 mM), or l-alanine (65 ± 10 mM).

### Stereochemistry of KDS formed by the *S. multivorum* SPT

**Figure 2:**
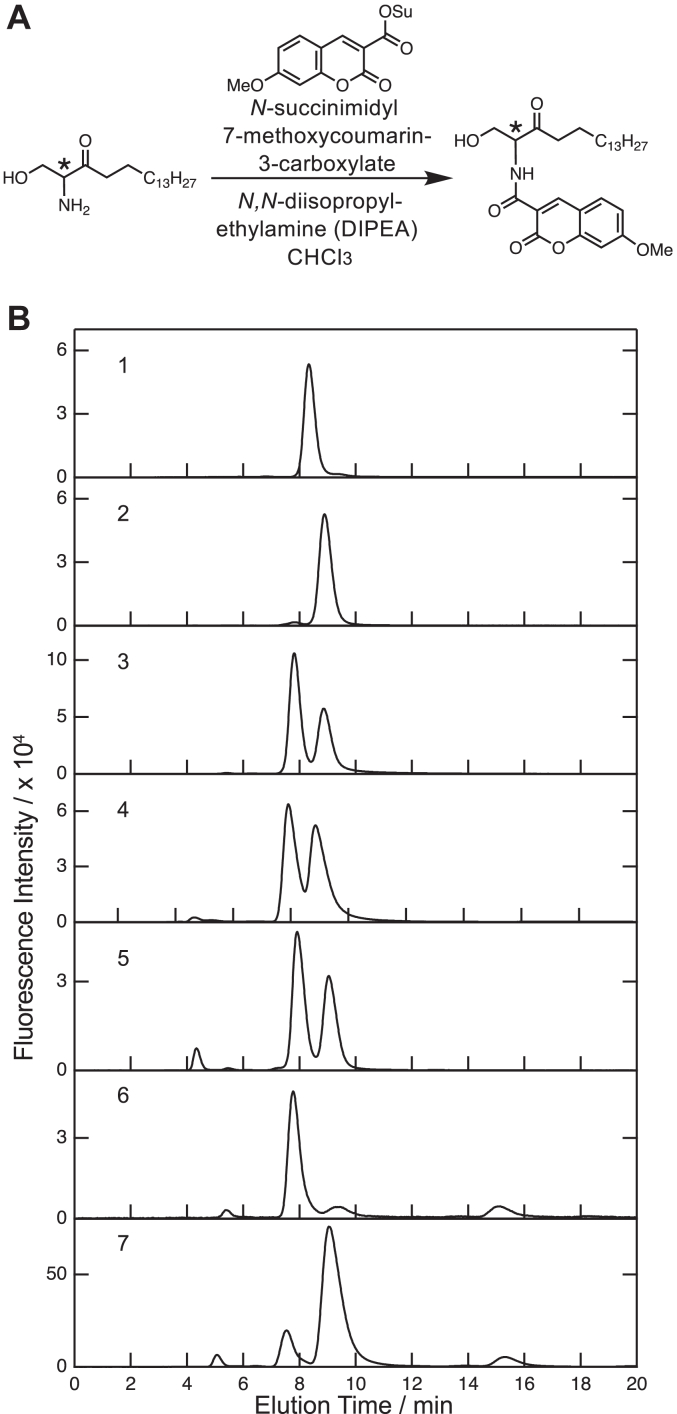
**Stereochemistry of KDS formed by the *Sphingobacterium multivorum* SPT.***A*, scheme of fluorescence derivatization of KDS enantiomers. The amino group of KDS was site specifically labeled to form (*S* or *R*)-*N*-(1-hydroxy-3-oxooctadecan-2-yl)-7-methoxycoumarin by using *N*-succinimidyl 7-methoxy-2-oxo-2*H*-chromene-3-carboxylate and DIPEA. *B*, chiral HPLC profiles of fluorescence-derivatized KDS. The *vertical axis* shows the fluorescence intensity, and the *horizontal axis* shows the retention time. *Line 1*, 7-methoxycoumarin-labeled l-serine–type (*2S*)-KDS; *line 2*, 7-methoxycoumarin–labeled d-serine–type (*2R*)-KDS; *line 3*, mixture of 7-methoxycoumarin–labeled l-/d-serine–type (*2S*/*2R*)-KDS; *lines 4 and 5*, 7-methoxycoumarin–labeled products from the reaction of 230 μM SPT with 100 mM l-serine and d-serine, respectively, and 1 mM palmitoyl-CoA at 37 °C for 120 min; and *lines 6 and 7*, 7-methoxycoumarin–labeled (*2S*)-KDS and (*2R*)-KDS (1.3 mM), respectively, after incubation with 230 μM SPT at 37 °C for 120 min. DIPEA, N, N-diisopropylethylamine; KDS, 3-ketodihydrosphingosine; PLP, pyridoxal-5′-phosphate; SPT, serine palmitoyltransferase.

KDS has a chiral center at the C2 position to which hydrogen, hydroxymethyl, amino, and acyl groups are attached. In the present study, it was necessary to know which optical isomer, (*2S*)- or (*2R*)-KDS, was formed from d-serine. In order to detect KDS enantiomers, we developed a new method in which each enantiomer of 7-methoxycoumarin-labeled KDS was separated and quantified by chiral HPLC (Fig. 2*A*). First, the standard compounds (*2S*)-2-amino-1-hydroxyoctadecan-3-one (l-serine–type (*2S*)-KDS) and (*2R*)-2-amino-1-hydroxyoctadecan-3-one (d-serine–type (*2R*)-KDS) were chemically synthesized (Supporting information). Next, the conditions for fluorescence labeling and HPLC were examined using these authentic compounds; the successful separation of KDS enantiomers was achieved by 7-methoxycoumarin labeling of the amino group of KDS (Fig. 2*B*, *lines 1–3*). The labeled (*2S*)-KDS and (*2R*)-KDS were eluted at 7.7 and 8.9 min, respectively, with the calculated separation factor of 1.3 (Fig. 2*B*, *line 3*). When the SPT reaction product derived from l-serine was analyzed, two fluorescence peaks were detected (Fig. 2*B*, *line 4*). The first peak corresponded to 7-methoxycoumarin-labeled (*2S*)-KDS and the second to 7-methoxycoumarin-labeled (*2R*)-KDS. Similarly, for the SPT reaction product derived from d-serine, two fluorescence peaks corresponding to 7-methoxycoumarin-labeled (*2S*)-KDS and 7-methoxycoumarin-labeled (*2R*)-KDS were detected (Fig. 2*B*, *line 5*). The areas of the two fluorescence peaks in each case were measured, and it was determined that (*2S*)-KDS and (*2R*)-KDS were present at a ratio of approximately 1:1 in both cases.

Then, we incubated (*2S*)-KDS or (*2R*)-KDS with SPT and analyzed whether racemization of each KDS enantiomer proceeded in an SPT-dependent manner. It was confirmed that the racemized product was formed in each case, albeit in a smaller amount (Fig. 2*B*, *lines 6* and *7*).

### α-Hydrogen exchange of serine by the *S. multivorum* SPT

**Figure 3:**
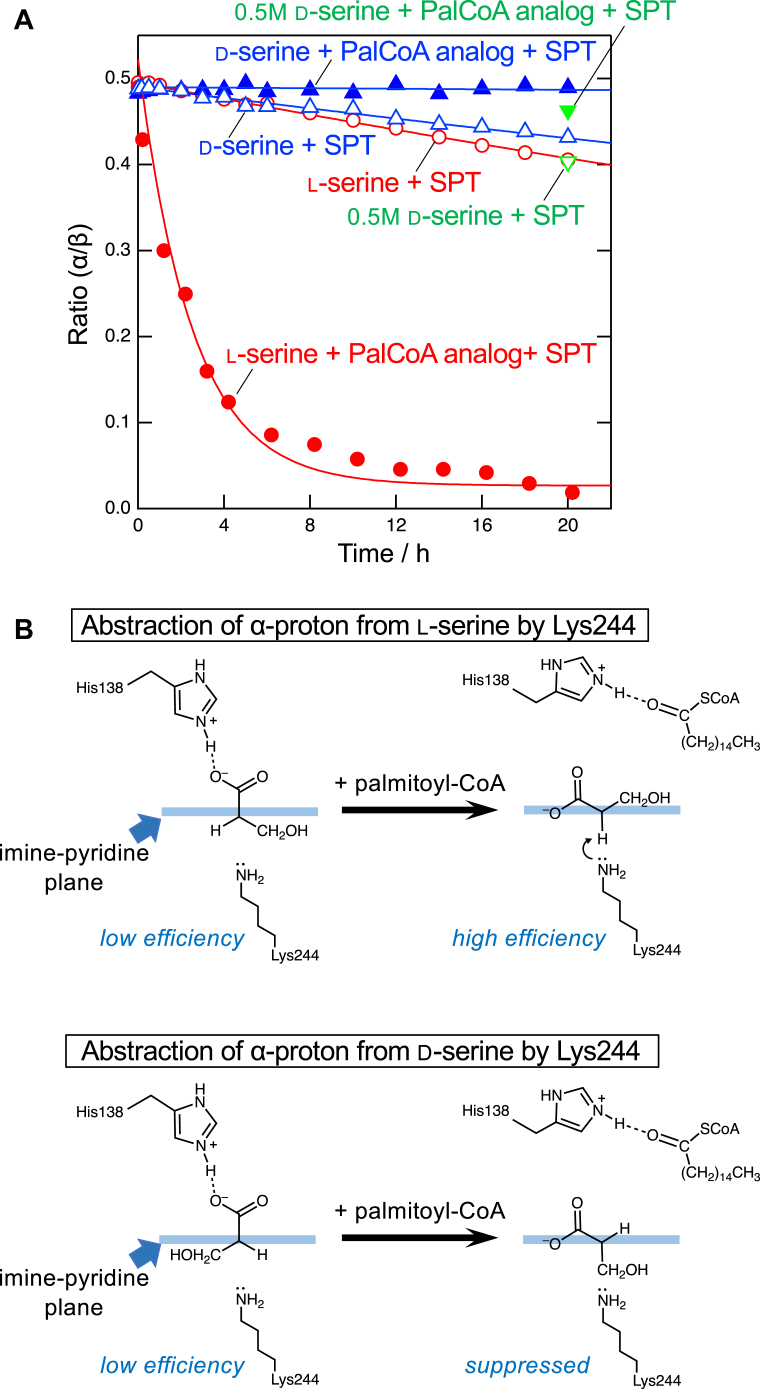
**SPT-dependent hydrogen-deuterium exchange at Cα of l- and D-serine.***A*, ratios of the integrated intensity of the NMR signals of α-hydrogen to that of β-hydrogens are plotted against the reaction time. The *lines* represent theoretical fits to a single-exponential decay equation. *Open circles*, 10 mM l-serine was incubated with 10 μM SPT; *closed circles*, 10 mM l-serine was incubated with 10 μM SPT and 1.5 mM *S*-(2-oxoheptadecyl)-CoA; *open triangles*, 10 mM d-serine was incubated with 10 μM SPT; *closed triangles*, 10 mM d-serine was incubated with 10 μM SPT and 1.5 mM *S*-(2-oxoheptadecyl)-CoA; *inverted open triangle*, 500 mM d-serine was incubated with 10 μM SPT for 20 h; and *inverted closed triangle*, 500 mM d-serine was incubated with 10 μM SPT and 1.5 mM *S*-(2-oxoheptadecyl)-CoA for 20 h. *B*, *schematic drawing* of the abstraction of α-proton from serine enantiomers, supported by crystal structures, is shown in the orientation viewed from C4 of PLP. Lys244 would withdraw the Cα hydrogen from d-serine or l-serine, immediately exchange it for deuterium from bulk D_2_O and subsequently add the deuterium to Cα of the quinonoid intermediate from the same side. PLP, pyridoxal-5′-phosphate; SPT, serine palmitoyltransferase.

The extent of the SPT-catalyzed hydrogen–deuterium exchange at Cα of d-serine was measured by ^1^H-NMR in the absence of the other substrate palmitoyl-CoA, to know whether the α-hydrogen is abstracted from d-serine by the *S. multivorum* SPT. In a D_2_O buffer at pD 7.5, both l-serine and d-serine gave the same NMR spectra showing four resonance peaks centered at 3.85 p.p.m. and eight resonance peaks around 3.98 p.p.m., corresponding to the α- and β-hydrogens, respectively (47). The hydrogen–deuterium exchange at Cα was observed by following the relative intensities of the α- and β-hydrogens as shown in Figure 3*A*.

No decrease in the α/β ratio was observed in the control experiments without SPT. Consistent with the previous observation (17), when 10 mM l-serine was incubated with 10 μM SPT, a slow exchange of the α-hydrogen for deuterium was observed (*t*_1/2_ = 67 ± 5.2 h), and, when 1.5 mM *S*-(2-oxoheptadecyl)-CoA was added to this l-serine–SPT reaction solution, the rate of the exchange was significantly increased (*t*_1/2_ = 1.7 ± 0.18 h), indicating that binding of the palmitoyl-CoA analog to SPT enhanced the α-hydrogen abstraction of l-serine. Similar spectral changes were observed when 10 mM d-serine was incubated with 10 μM SPT, although the exchange of the α-hydrogen was slightly slower than that for l-serine. However, the α-hydrogen exchange of d-serine was almost suppressed in the presence of 1.5 mM *S*-(2-oxoheptadecyl)-CoA. These results on the reactions of the *S. multivorum* SPT with d-serine are consistent with the observations for the *S. paucimobillis* SPT (47). Incubation with a higher concentration, 500 mM, of d-serine slightly, but significantly, increased the rates of the hydrogen–deuterium exchange at Cα in both the absence and presence of *S*-(2-oxoheptadecyl)-CoA as shown in Figure 3*A*.

### Serine racemase activity of the *S. multivorum* SPT

**Figure 4:**
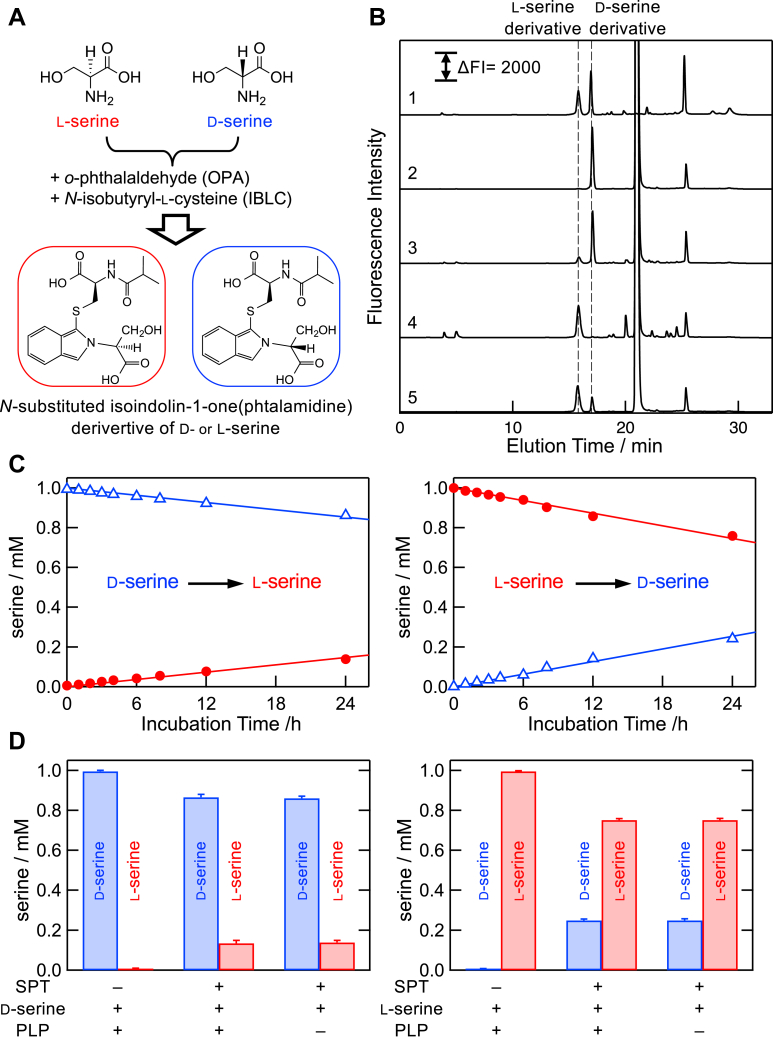
**Serine racemase activity of the *Sphingobacterium multivorum* SPT.***A*, scheme of the fluorescence derivatization procedure for l- and d-serine. *B*, HPLC profiles of fluorescence-derivatized serine enantiomers of authentic compounds and enzyme-catalyzed products. The *vertical axis* shows the fluorescence intensity, and the *horizontal axis* shows the retention time. One millimolar l-serine or d-serine was incubated with or without 47 μM SPT at 37 °C for 24 h. *Line 1*, mixture of 0.01 mM each of authentic fluorescence-derivatized l-serine and d-serine; *line 2*, reaction mixture of d-serine without SPT; *line 3*, reaction mixture of d-serine with SPT; *line 4*, reaction mixture of l-serine without SPT; and *line 5*, reaction mixture of l-serine with SPT. *C*, time-dependent racemization from d-serine (*left*) and l-serine (*right*) with SPT. The reaction mixture containing 1 mM serine and 47 μM SPT was incubated at 37 °C for 1 to 24 h. *D*, SPT-dependent formation of l-serine from d-serine (*left*) and d-serine from l-serine (*right*) after incubation at 37 °C for 24 h. The reaction mixture contained 1 mM serine and either or both of 1 mM PLP and 47 μM SPT. PLP, pyridoxal-5′-phosphate; SPT, serine palmitoyltransferase.

In order to verify the possibility of conversion between d-serine and l-serine, the serine racemase activity of SPT in the absence of palmitoyl-CoA was examined by the combination of fluorescence labeling and reverse-phase HPLC (Fig. 4). As shown in an HPLC profile in Figure 4*B* (*line 1*), serine enantiomers were successfully separated by reverse-phase HPLC after the modification of serine with o-phthalaldehyde and *N*-isobutyryl-l-cysteine as indicated in Figure 4*A* (48). Based on the fluorescence intensity of the modified authentic serine, the purities of d- and l-serine used in this study were 99.6% and 99.9%, respectively (Fig. 4*B*, *lines 2* and *4*). After the incubation of d-serine with SPT, l-serine was clearly detected in the assay mixture (Fig. 4*B*, *line 3*). Alternatively, when l-serine was incubated with SPT, d-serine was also produced in the assay mixture (Fig. 4*B*, *line 5*). As shown in Figure 4*C*, time-dependent racemization of serine was observed with 1 mM d-serine or l-serine and 47 μM SPT. The formation rate of l-serine from d-serine was 0.0020 min^–1^. This value was comparable to the apparent catalytic efficiency ($k_{cat}^{app}/K_{m}^{app}$) of KDS formation from d-serine (0.0013 min^–1^ mM^–1^) (Table 1). As shown in Figure 4*D*, the racemization of serine was observed only in the presence of SPT, and free PLP (1 mM) did not catalyze the racemization reaction. Racemase activity of SPT is orders of magnitude lower than that of other known serine racemases (49, 50, 51).

### Crystal structure of the *S. multivorum* SPT after soaking in a d-serine solution

**Figure 5:**
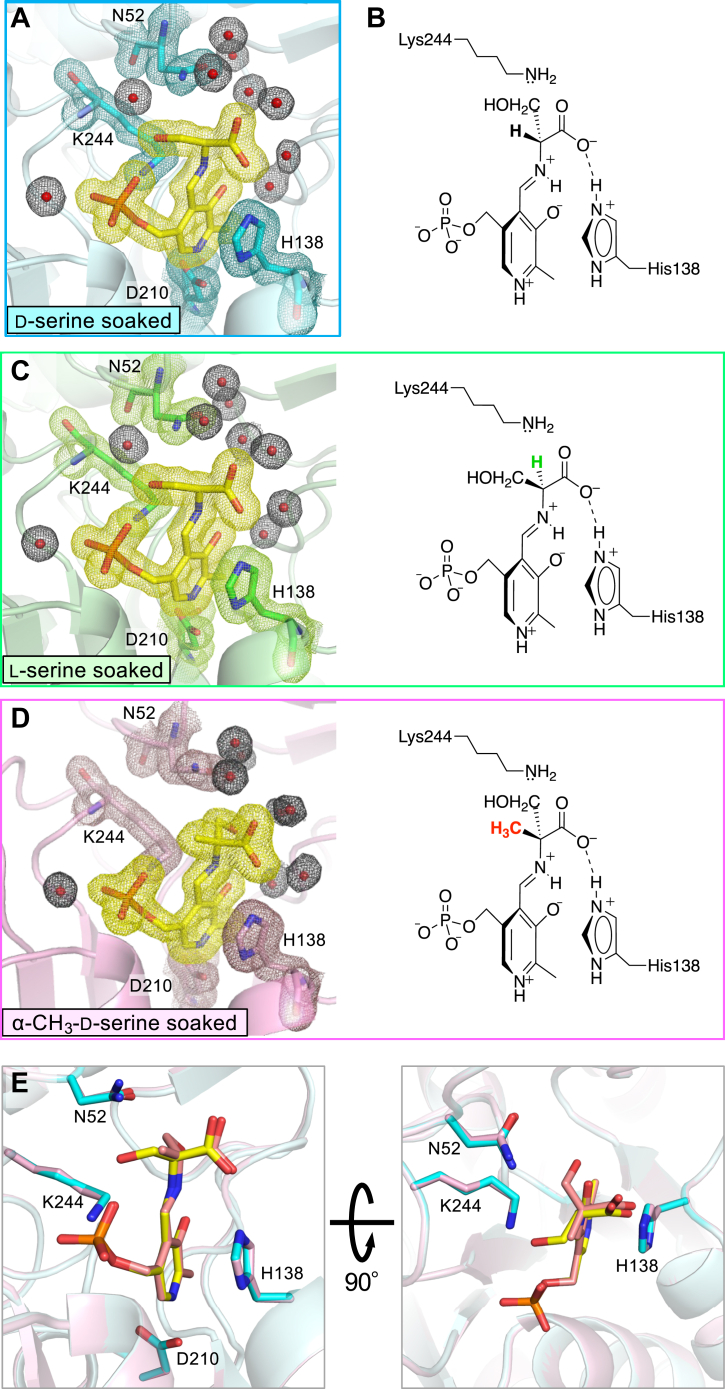
**Active site of the *Sphingobacterium multivorum* SPT obtained by soaking the crystal with d-serine or α-methyl-d-serine.***A*, *close-up view* of the active site of the d-serine–soaked SPT crystal. *B, schematic representation* of the expected PLP–d-serine external aldimine intermediate. *C*, *close-up view* of the active site of the l-serine–soaked SPT crystal forming the PLP–l-serine external aldimine intermediate (PDB: 8H1Q) (*left*) and its schematic representation (*right*). *D, close-up view* of the active site of the α-methyl-d-serine–soaked SPT crystal forming the PLP–α-methyl-d-serine external aldimine intermediate (*left*) and its schematic representation (*right*). The amino acid residues, Asn52, His138, Asp210, and Lys244, and the PLP external aldimine moiety are shown in *stick representation*. The carbon atoms of the aldimine moiety are color coded by *yellow* and those of the polypeptides for each structure are by *cyan* (*A*), *green* (*C*), and *magenta* (*D*), and their nitrogen, oxygen, and phosphorus atoms are colored *blue*, *red*, and *orange*, respectively. Water molecules are drawn as *spheres*. Calculated 2*F*o–*F*c omit electron density map contoured at 1 σ level is shown for the PLP–amino acid external aldimine, water molecules, and the four amino acid residues by *meshes*. *E*, overlay of the active sites of the SPT–l-serine complex prepared by d-serine soaking (*yellow* and *cyan*) and the SPT–α-methyl-d-serine complex (*pale orange* and *light magenta*). PLP, pyridoxal-5′-phosphate; SPT, serine palmitoyltransferase.

To further study the substrate recognition mechanism and the mechanism of the KDS production from d-serine by SPT, we tried to prepare the crystal of SPT complexed with d-serine by a soaking experiment. The SPT crystals were soaked in a solution supplemented with 190 mM d-serine for 10 min and subjected to X-ray structure analysis. This d-serine–soaked crystal diffracted to 1.65 Å resolution, and the diffraction data were indexed in the tetragonal space group *P*4_1_2_1_2 with unit cell parameters, a = b = 61.0 Å, c = 207.3 Å, α = β = γ = 90°. A summary of the data statistics is shown in Table 2. The crystal structure was determined at a resolution of 1.65 Å and refined to *R*_work_ and *R*_free_ values of 0.149 and 0.184, respectively, with a Cruickshank diffraction-component precision index of 0.0921 Å. The refinement statistics are also summarized in Table 2. The final model contained 394 amino acid residues, one PLP, 419 water molecules, and seven ethylene glycol molecules per monomer. The model contained 394 of 398 amino acid residues from Ser2 to Val395, and the last three C-terminal residues had no defined electron density, consistent with observations for other SPT–amino acid complexes reported previously (23). The active-site structure of d-serine–soaked SPT is shown in Figure 5*A*. While the 2*F*o-*F*c electron density map calculated from the dataset clearly showed the formation of a PLP–amino acid external aldimine intermediate (Fig. 5*A*), a model of the PLP–d-serine external aldimine could not fit this electron density map (refer the schematic drawing of the proposed PLP–d-serine external aldimine in Fig. 5*B*). Unexpectedly, a model of the PLP–l-serine external aldimine was well assigned at the occupancy of 1 to this electron density map. The RMSD of the d-serine–soaked crystal structure and the l-serine complex structure (PDB 8H1Q) superimposed by the least-squares method between the Cα atoms was 0.14 Å, indicating that there was little change in the overall structure between the d-serine–soaked crystal and the l-serine–soaked crystal. In the two crystals, the architecture of the active site surrounding the PLP–l-serine external aldimine, that is, orientations of the side chains of amino acid residues, numbers and positions of water molecules, and hydrogen-bond networks, was exactly the same (Fig. 5, *A* and *C*).

**Table 2:** Data collection and refinement statistics

Parameter	Value(s) for:	
	l-serine complex formed by d-serine soaking	d-methyl–serine complex
Data collection^a^		
Diffraction source	SPring-8 BL38B1	PF BL17A
Wavelength (Å)	0.9	0.98
Temperature (K)	100	100
Detector	Rayonix MX225HE	Dectris Eiger X16M
Crystal-detector distance (mm)	200	180
Rotation range per image (°)	0.2	0.25
Total rotation range (°)	180	135
Exposure time per image (s)	1.0	0.5
Space group	*P*4_1_2_1_2	*P*4_1_2_1_2
Cell dimensions		
a, b, c (Å)	61.0, 61.0, 207.3	61.8, 61.8, 208.3
α, β, γ (°)	90, 90, 90	90, 90, 90
Resolution range (Å)	50.0–1.65 (1.75–1.65)	50–1.70 (1.80–1.70)
⟨ *I*/σ(*I*)⟩	38.7 (9.7)	20.5 (3.8)
*R*_meas_^b^	0.043 (0.180)	0.07 (0.651)
*CC*_1/2_^c^	1.00 (0.991)	0.999 (0.959)
*R*_p.i.m._	0.013 (0.077)	0.024 (0.253)
Completeness (%)	99.2 (95.3)	97.5 (99.0)
Redundancy	12.9 (9.3)	10.1 (10.3)
Mosaicity (°)	0.177	0.097
Refinement^a^		
No. of reflections, working set	37,909 (365)	35,935 (279)
Resolution range (Å)	42.25–1.65 (1.69–1.65)	46.19–1.70 (1.74–1.70)
*R*_work_*/R*_free_^d^	0.149/0.184	0.173/0.219
Completeness (%)	99.2	97.5
Cruickshank DPI (Å)	0.0921	0.1147
No. of non-H atoms^e^		
Protein	3197	3173
PLP-external aldimine	22	23
Ethylene glycol	28	16
Water	419	387
RMSDs^f^		
Bonds (Å)	0.010	0.011
Angles (°)	1.682	1.706
Average *B* factors (Å^2^)^g^		
Protein	18.9	26.4
PLP-external aldimine	12.7	19.7
Ethylene glycol	41.2	54.6
Water	30.5	38.0
Ramachandran plot		
Most favored (%)	97	96
Allowed (%)	3	4
PDB ID	8IYP	8IYT

DPI, diffraction-component precision index; PLP, pyridoxal-5′-phosphate.

a One crystal was used for each dataset. Values in parentheses are for the highest resolution shell.

b *R*_mears_ = $\sum_{hkl}{\left[N/\left(N-1\right)\right]}^{1/2}\times \sum_{i}\left|I_{i}\left(hkl\right)-\left\langle I\left(hkl\right)\right\rangle \right|/\sum_{hkl}\sum_{i}I_{i}\left(hkl\right)$, where $I_{i}\left(hkl\right)$ is the intensity of the *i*th observation of reflection (hkl) and *N* is the redundancy.

c Values of the highest resolution shells. *CC*_1/2_, Pearson correlation calculated between two random half datasets.

d *R*_free_ was calculated as for *R*_cryst_, but is calculated for 10% of the reflections that were chosen at random and omitted from the refinement process (66).

e Corresponding two atoms of 50% occupancies in the double conformers were counted separately.

f RMSD.

g The average temperature factor was calculated based on the values of no. of non-H atoms.

### Crystal structure of the SPT–α-methyl-d-serine complex

The amount of l-serine in the commercial d-serine sample was below the detection limit, but the possibility that the binding affinity of d-serine to SPT in crystal is so low that even a trace amount of contaminating l-serine yields the SPT–l-serine binary complex cannot be ruled out. Therefore, to confirm that the d-form of serine really binds to SPT, we analyzed the crystal structure of the SPT–α-methyl-d-serine complex. If α-methyl-d-serine carrying a methyl group at the Cα position could bind to the SPT active site, it is likely that d-serine can also bind to SPT.

The *K*_d_ value for α-methyl-d-serine in solution was 27 ± 2.5 mM, while that for d-serine was 11 ± 1.8 mM (Fig. S1 and Table 1). The crystal of the binary complex of SPT with α-methyl-d-serine could be prepared by soaking the SPT crystal into the buffer supplemented with 185 mM α-methyl-d-serine for 40 min. The crystal structure was determined at a resolution of 1.70 Å and refined to *R*_work_ and *R*_free_ values of 0.173 and 0.219, respectively, with a Cruickshank diffraction-component precision index of 0.1147 Å. A summary of the data statistics is shown in Table 2. The final model contained 394 amino acid residues (Ser2 to Val395), one PLP, 384 water molecules, and four ethylene glycol molecules per monomer. The RMSD of the α-methyl-d-serine-soaked SPT structure and the l-serine–soaked SPT structure (8H1Q) was 0.15 Å, and that of the α-methyl-d-serine–soaked SPT structure and the d-serine–soaked SPT structure was 0.17 Å. These values indicate little change in the overall structures among the three SPT–amino acid complexes. Four amino acid residues in the SPT active site, Asn52, His138, Asp210, and Lys244, were selected as key residues and drawn as stick models in Figure 5. The conformations and orientations of the side chains of the three amino acid residues surrounding the PLP molecule, His138, Asp210, and Lys244, appear to be exactly the same (Fig. 5, *A*, *C*, and *D*).

The 2*F*o-*F*c electron density map calculated from the dataset clearly indicated the formation of the external aldimine intermediate (Fig. 5*D*). A model of the PLP–α-methyl-d-serine external aldimine could be well assigned at the occupancy of 1 to this electron density map. This result shows that the serine analog could bind to the active site of SPT in the crystal while keeping its d-configuration. The carboxy group of α-methyl-d-serine formed a hydrogen bond/ionic interaction with Nε2 of His138 as did the carboxy group of l-serine. Due to this fixation, the hydroxymethyl group of α-methyl-d-serine turned toward the O3′ atom of PLP, and its β-hydroxy group occupied the space that a water molecule occupied in the SPT–l-serine complex. The α-methyl group of α-methyl-d-serine was placed in the space occupied by the hydroxymethyl group of the SPT–l-serine complex. One water molecule that intermediated between the side chain of Asn52 and the hydroxymethyl group of l-serine *via* hydrogen bonds was expelled, and the orientation of the side-chain amide group of Asn52 was shifted by 0.8 Å (Fig. 5*E*).

## Discussion

**Figure 6:**
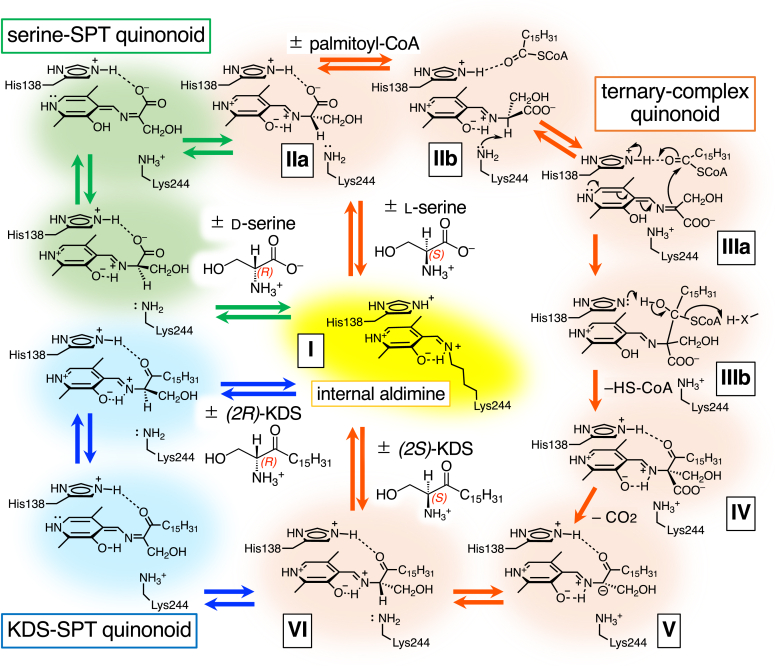
**Proposed reaction mechanism of the *Sphingobacterium multivorum* SPT.** At the active site of SPT, PLP forms an aldimine with the ε-amino group of Lys244 (internal aldimine, I, highlighted in *yellow*). The SPT reaction pathway (Ⅱ∼Ⅵ) with l-serine and palmitoyl-CoA is shown by *orange arrows* and highlighted in *orange*. The pathway of d-serine or KDS racemization is shown by *green* or *blue*, respectively. The binding of the first substrate l-serine leads to the PLP–l-serine external aldimine (Ⅱa) by transaldimination. As shown in the crystal structure of the SPT–l-serine binary complex (Fig. 5), the conformation of the external aldimine in this complex, caused by the hydrogen/ionic bond between the carboxy group of l-serine and Nε2 of His138, is unfavorable for α-deprotonation of l-serine. Binding of the second substrate palmitoyl-CoA induces changes in the orientation of the l-serine moiety of the external aldimine to cancel the interaction with the side chain of His138, and the carbonyl group of palmitoyl-CoA binds to Nε2 of His138 (Ⅱb). This causes the Cα–H bond to be nearly perpendicular to the imine-pyridine plane, which is favorable for α-deprotonation to form the quinonoid intermediate (ternary-complex, Ⅲa). The carbanionic Cα of Ⅲa attacks palmitoyl-CoA (Claisen-type condensation), and CoA-SH is released from Ⅲb to form a condensation product (Ⅳ), which by decarboxylation yields the second quinonoid intermediate (Ⅴ). Protonation at Cα of V yields the external aldimine of PLP–*(2S)*-KDS (Ⅵ). Finally, the release of *(2S)*-KDS regenerates the internal aldimine (I). d-Serine or *(2R)*-KDS can also bind to the SPT active site to form the corresponding external aldimine intermediate. In both cases, racemization occurs *via* the quinonoid intermediates, and the ε-amino group of Lys244 is thought to be responsible for the hydrogen transfer at the C2 position. KDS, 3-ketodihydrosphingosine; PLP, pyridoxal-5′-phosphate; SPT, serine palmitoyltransferase.

We have proposed a seven-step reaction mechanism where SPT produces KDS from l-serine and palmitoyl-CoA starting from the internal aldimine (I) formed between PLP and the ε-amino group of Lys244 at the active site of SPT (Fig. 6): (1) the external aldimine (IIa) formation between PLP and the first substrate l-serine *via* transaldimination; (2) the ternary complex (IIb) formation after the second substrate palmitoyl-CoA binds to the external aldimine; (3) the quinonoid intermediate (IIIa) formation by the deprotonation at the Cα position of the l-serine moiety of IIb by Lys244; (4) the condensation product (IV) formation by the nucleophilic attack of the quinonoid Cα (IIIa) on the palmitoyl-CoA thioester (Claisen-type condensation) and subsequent release of CoA-SH; (5) the carbanion intermediate (V) formation by decarboxylation of IV; (6) the external aldimine of PLP–KDS (VI) formation by the protonation at Cα of V; and finally, (7) the regeneration of the internal aldimine (I) by release of KDS.

Although our previous biochemical and structural findings showed that microbial SPTs have a relatively broad substrate specificity for both amino acids and acyl-CoA (15, 16, 23), the detection of KDS after the incubation of d-serine and palmitoyl-CoA with SPT was an unexpected result. Consistent with the previous observation for the *S. paucimobillis* SPT, ^1^H-NMR analysis showed that Cα of both l-serine and d-serine was slowly deprotonated in the active site of the *S. multivorum* SPT (Fig. 3*A*). When a palmityl-CoA analog coexisted, the α-deprotonation was significantly accelerated for l-serine but almost suppressed for d-serine. This could be explained by the stereochemistry around Cα in the ternary complex (Fig. 3*B*): the position of the α-hydrogen of the l-serine–SPT binary complex is not suitable for the proton withdrawal by Lys244 as confirmed by the crystal structure, and, upon binding palmitoyl-CoA, the serine moiety of the external aldimine would rotate to the conformation with its α-hydrogen perpendicular to the pyridine ring of PLP, which is ideal for the proton withdrawal. In the case of d-serine, the same rotation caused by palmitoyl-CoA binding should position the α-hydrogen not only in an oblique angle relative to the pyridine ring of PLP but also away from Lys244. But, at a much higher concentration of d-serine, 500 mM, which is the same as that in the KDS formation assay (Fig. 1), the deprotonation was not completely suppressed by the palmitoyl-CoA analog (Fig. 3*A*). These results indicate that the quinonoid intermediate, which adopts a planar conformation and loses the stereochemistry of serine, is tentatively produced to some extent. Then, l-serine, the original substrate of SPT, can be formed by reprotonation of the quinonoid intermediate derived from d-serine. This racemization reaction was confirmed by the fluorescence labeling of serine and HPLC analysis (Fig. 4). The rates for the SPT-catalyzed α-deprotonation and racemization of d-serine were comparable to that for the catalytic efficiency of KDS formation from d-serine.

The crystal structure of d-serine–soaked SPT solved at a 1.7Å resolution showed that l-serine formed by racemization of d-serine was trapped in the SPT active site as the PLP–l-serine external aldimine intermediate (Fig. 5*A*). The SPT crystal of the binary complex with α-methyl-d-serine, which has a bulkier side chain than that of d-serine and has less than half the binding affinity to SPT than d-serine, showed the clear electron density of the PLP–α-methyl-d-serine external aldimine, suggesting that the active site of SPT can also accommodate d-serine and that this crystal structure mimics the SPT–d-serine binary complex preceding the racemization reaction. The reason why the electron density of the PLP–d-serine external aldimine was not detected in the d-serine–soaked SPT crystal is explained as follows: d-serine was converted to l-serine by SPT in the crystal during the soaking treatment, and the l-serine with more than 20 times higher affinity to SPT than d-serine remained in the active site, resulting in the structure of the l-serine complex of SPT.

It was surprising that both of the two KDS enantiomers were detected in the SPT reaction product from l-serine or d-serine and palmitoyl-CoA (Fig. 2, *lines 4* and *5*). It was also found that SPT could catalyze the racemization of KDS (Fig. 2, *lines 6* and *7*). In the latter racemization reaction, KDS was added to the SPT solution, and it is likely that only a small portion of the hydrophobic KDS added to the aqueous solution actually binds to SPT without sticking to the tube wall. On the other hand, in the former KDS formation reaction, KDS is formed in the SPT active site. The KDS thus generated would not easily dissociate from SPT in the aqueous solution and racemization proceeds while remaining in the active site of SPT. This might explain the difference in the extent of racemization between the two experiments: KDS was completely racemized to the 1:1 ratio in the KDS formation experiment, while only partially in the KDS racemization experiment. Under physiological conditions, KDS would be immediately transferred to KDS reductase, the next enzyme in the biosynthetic pathway of sphingolipids, thus preventing the racemization of KDS.

Based on the enzymological and structural results reported here, it is thought that SPT does not catalyze the decarboxylative condensation between d-serine and palmitoyl-CoA but that d-serine is racemized by SPT to l-serine *via* the quinonoid intermediate (Fig. 6, green arrows). The resultant l-serine is then utilized as the substrate of SPT to from (*2S*)-KDS. However, palmitoyl-CoA would effectively inhibit the racemization at physiological concentrations of d-serine, although the α-deprotonation of d-serine could be detected with a high concentration, 500 mM, of d-serine even in the presence of the palmitoyl-CoA analog.

As for the racemization of KDS by SPT (Fig. 6, blue arrows), its physiological significance would also be negligible: *(2S)*-KDS formed by SPT from l-serine is metabolized by KDS reductase to d-*erythro (2S,3R)*-sphinganine and *(2R)*-sphinganine has not been detected in biological samples. Since sphinganine contains two chiral centers at the C2 and C3 positions, there are three additional nonnatural stereoisomers of sphinganine. The synthetic l-*threo* (*2S, 3S*)-stereoisomer of natural d-*erythro*-sphinganine, called Safingol, is known to be an inhibitor of PKC and sphingosine kinase (52, 53) and has been shown in several human phase 1 clinical trials to act synergistically with other chemotherapeutic agents by potentiating chemotherapy drug–induced apoptosis as well as to induce autophagy in Safingol monotherapy (54, 55, 56). Interestingly, Safingol is known to be recognized and metabolized by enzymes of the sphingolipid biosynthetic pathway and converted into l-*threo* dihydroceramide or glycosphingolipids despite its nonnatural stereochemistry (57, 58). Although the remaining two stereoisomers, that is, d-*threo* (*2R, 3R*)-sphinganine and l-*erythro* (*2R, 3S*)-sphinganine, have not been detected in biological samples, they might have some biological functions or toxicity. It is also interesting to see if (*2R*)-KDS, which can be efficiently synthesized using SPT from l-serine and palmitoyl-CoA as shown in the present study, becomes a substrate for KDS reductase and is converted to d-*threo* (*2R, 3R*)-sphinganine.

## Experimental procedures

### Expression and purification of the *S. multivolum* SPT

Expression and purification of *the S. multivolum* SPT were performed as previously reported (16, 17). Briefly, *Escherichia coli* BL21 (DE3) pLysS cells (Novagen) harboring the plasmid expressing the full-length SPT (16) were grown, and SPT expression was induced with 0.1 mM IPTG at 37 °C for 4 h. After harvesting, *E. coli* cells were sonicated in 50 mM Tris–HCl buffer (pH 7.5) with 0.1 mM EDTA and centrifuged to prepare the crude extract. The SPT was purified using HiPrep DEAE-FF crude 16/10, HiPrep butyl-FF crude 16/10, and the second HiPrep DEAE-FF crude 16/10 in an ÄKTA FPLC system (GE Healthcare). The purified SPT was desalted to 20 mM potassium phosphate buffer (pH 7.4) containing 0.1 mM EDTA and concentrated to 20 mg/ml and then stored at 4 °C. The concentration of the purified SPT subunit in the solution was determined spectrophotometrically (using the molar extinction coefficient of 26,780 M^–1^ cm^–1^ at 280 nm for the PLP form of the enzyme) (16).

### Synthesis of authentic standards of l-Ser– and d-Ser–type KDSs

Chemical synthesis of (2*S*)-2-amino-1-hydroxyoctadecan-3-one (l-serine–type KDS) and (2*R*)-2-amino-1-hydroxyoctadecan-3-one (d-serine–type KDS) was done using commercially available materials based on the method previously reported (59). For details of the synthesis, see Supporting information.

### TLC analysis of KDS

The assay was performed as previously reported (23). For qualitative analysis, 500 mM of l-serine or d-serine was incubated with 1 mM palmitoyl-CoA and 100 μM SPT in 100 μl of 100 mM potassium phosphate, pH 7.5, at 37 °C for 60 min. [1-^14^C] palmitoyl-CoA (20 kBq/μmol) was supplemented into the above reaction mixture as the tracer. The reactions were terminated by addition of the equal volume of 2 N ammonia. Lipids were extracted by successive addition and mixing of 750 μl of chloroform/methanol (1:2, v/v), 250 μl of chloroform, and 250 μl of 1% KCl. Phases were separated by centrifugation, and the organic phase was recovered. The aqueous phase was re-extracted with 200 μl of chloroform, and, after centrifugation, the organic phase was combined with the organic phase from the first extraction. The organic phase was washed with water/chloroform/methanol (47:48:3, v/v), dried, and suspended in chloroform/methanol (2:1, v/v). The extracted lipids were resolved by normal-phase TLC on Silica Gel 60 high-performance TLC plates (Merck) with chloroform/methanol/2 N ammonia (40:10:1, v/v) or chloroform/methanol/triethanol amine (95:5:10, v/v) and visualized by spraying with ninhydrin reagent followed by gentle heating. Radioactive lipids on TLC plates were visualized by using a BAS2000 Image Analyzer (Fujifilm). For the quantitative analysis, the UV-fluorescence intensities of the reaction products on TLC plates were visualized by a Fusion chemiluminescence imaging system using a UV-light box and analyzed by a FUSION Capt software (Vilber Lourmat).

### LC-MS/MS analysis of the reaction products

Liquid chromatography (HPLC) was performed on an LC system Nexera X2 (SHIMADZU) equipped with l-Column ODS (3 μm, 3 × 150 mm) (CELI). The HPLC mobile phases were as follows: A1, methanol/water (60:40, v/v); B1, methanol containing 0.1% formic acid, and 5 mM ammonium formate. A gradient program was used for the HPLC separation at a flow rate of 0.2 ml/min; the program consisted of holding at 40% B1 for 6 min, linearly increasing to 100% B1 over 10 min, and keeping it for 5 min, followed by re-equilibration to the initial condition for 35 min. The HPLC system was connected to a 4500 QTRAP hybrid triple quadrupole/linear ion trap mass spectrometer (AB SCIEX) equipped with a Turbo Ion Spray source. The mass spectrometer was run in the positive mode with the curtain gas set at 10 and interface heater “on.” Ion spray voltage was used with an optimal value for analysis of a sphingoid base, 5500 V. Parameters of the temperature, ion source gas 1 (GS1), and GS2 were set at 600 °C, 80 psi, and 45 psi, respectively. Identification of structure-specific fragments was performed in product ion scan mode. Data acquisition and analysis were performed using Analyst Software version 1.4.1 (AB SCIEX).

### Preparation of 7-methoxycoumarin-labeled KDS enantiomers

Fluorescence labeling of each KDS enantiomer (21 mg in 5.0 ml of chloroform) was performed by mixing with 17 mg of N-succinimidyl-7-methoxycoumarin-3-carboxylate and 11 μl of N, N-diisopropylethylamine under shielded light and stirring at room temperature for 2 h, and 7-methoxycoumarin-labeled KDS was obtained in an average yield of 87% for l-serine type (22 mg) or 83% for d-serine type (18 mg). The specific rotation [α]_D_ of each compound was measured as follows: +13.5 for 7-methoxycoumarin-labeled l-serine–type KDS and −12 for 7-methoxycoumarin-labeled d-serine–type KDS (c 0.5; CHCl_3_). The labeled KDS sample was then washed with diethyl ether, in which KDS is insoluble, to remove impurities from the reaction mixture.

### 7-Methoxycoumarin labeling of the reaction product derived from l-/d-serine by SPT

l-serine or d-serine (100 mM) and palmitoyl-CoA (1 mM) were incubated with 230 μM SPT in 100 mM potassium phosphate, pH 7.5, at 37 °C for 120 min. The reaction products were extracted and dried as described above for the TLC assay. *N*-succinimidyl 7-methoxycoumarin-3-carboxylate and N, N-diisopropylethylamine/chloroform was added to the reaction products and kept at room temperature. After 1 h, the precipitate was filtered off, the filtrate was dried on a rotary evaporator, and then the crude residue was washed with diethyl ether and the 7-methoxycoumarin-labeled product was obtained without further purification.

### HPLC analysis of fluorescence-labeled KDS enantiomers

The enantioselective detection of 7-methoxycoumarin-labeled KDS was performed on an HPLC system with a Jasco PU-2089 Plus pump equipped with a Jasco FP-2020 Plus fluorescence detector (JASCO Corporation). The labeled product was injected into a chiral column (CHIRALPAK IB, DAICEL corporation, 4.6 i.d. × 250 mm). Flow rate and column temperature were 1 ml/min and 37 °C. For fluorescence detection, the excitation and emission wavelengths were 380 nm and 450 nm, respectively. Composition of the mobile phase was 2-propanol:hexane:chloroform = 47.5:47.5:5 (v/v). Injection volume was 5 μl, containing 0.025 μmol of 7-methoxycoumarin-labeled KDS.

The separation coefficient α was calculated as follows:

$$k_{1}=\frac{t_{1}-t_{0}}{t_{0}},\;k_{2}=\frac{t_{2}-t_{0}}{t_{0}},\;\alpha =\frac{k_{2}}{k_{1}}$$

In these equations, k_1_ and k_2_ (k_1_ < k_2_) are the retention factors of the two peaks, t_1_ and t_2_ (t_1_ < t_2_) are the retention times of the two peaks, and t_0_ is the holdup time at which the sample begins to pass through the column. The calculated value, α = 1.3, confirms adequate separation of the 7-methoxycoumarin–labeled KDS enantiomers.

### Kinetic analysis of SPT for d-serine

Steady-state kinetic parameters were determined by varying the concentrations of d-serine in the presence of 1 mM palmitoyl-CoA in 100 μl of 100 mM potassium phosphate, pH 7.5, at 37 °C. The concentration of SPT and the incubation time were 10 μM and 20 min. The range of concentrations of d-serine was 0 to 680 mM. The reaction termination, lipid extraction, TLC analysis, and quantification of the reaction products on TLC plates were described above. The apparent velocities (min^–1^) *versus* amino acid concentrations (mM) plots were fitted to the Michaelis–Menten equation, v = *V*_max_[S]/(*K*_m_+[S]), by nonlinear regression using the Igor Pro 6.37 software (Wave Matrix Inc; https://www.wavemetrics.com) to determine the apparent kinetic parameters, $k_{cat}^{app}$ and $K_{m}^{app}$, of SPT for d-serine.

### Serine racemase assay of SPT

Serine racemase assay of SPT was performed by determining the amount of each antipodal serine derivative formed from d-serine or l-serine as previously reported with some modifications (48). After d/l-serine was derivatized to fluorescent diastereomer, the enantioselective detection of serine was performed on an HPLC system with a Multi lambda Fluorescence Detector (Waters Corporation). The reaction was carried out at 37 °C for 1 to 24 h in a reaction mixture (100 μl) containing 100 mM Tris/HCl buffer (pH 7.5), 1 mM d/l-serine, and 47 μM SPT with or without 1 mM PLP. After incubation, the reaction mixture was mixed successively with 100 μl of 0.1 N HCl and 800 μl of 200 mM sodium borate buffer (pH 10) containing 1.75 mg/ml *N*-isobutyryl-l-cysteine and 1.25 mg/ml *o*-phthalaldehyde. The mixture was diluted to one-tenthand injected into the reverse-phase HPLC column (COSMOSIL 5C18-AR-II, 4.6 mm i.d. × 250 mm, Nacalai Tesq. Inc). Flow rate and column temperature were 1 ml/min and 37 °C. For fluorescence detection, the excitation and emission wavelengths were 350 nm and 450 nm, respectively. Elution gradient condition was applied using the following eluents: eluent A, 10 mM sodium-borate/10 mM sodium phosphate buffer (pH 8.2); eluent B, methanol/acetonitrile/eluent A/water (40:40:10:10, v/v). The gradient program was as follows: 15% B for 11 min; 15% to 60% B for 12 min; 60% B for 3 min; 60% to 15% B for 2 min; 15% B for 7 min. The derivatized l-serine and d-serine were used as calibration standards (concentration range of 0.001–0.05 mM).

### NMR measurements

For the NMR spectroscopy, all exchangeable hydrogens of the enzyme, the ligands, and the buffer base were replaced with deuterium by a previously described method (47). Potassium pyrophosphate buffer (50 mM, pD 7.5) prepared in D_2_O was used. The ^1^H NMR spectra were measured in Wilmad 5-mm NMR tubes kept at 25 °C using a Bruker Avance III HD 600 MHz operating at 600 MHz. A flip angle of 30 °C with a relaxation delay time of 5 s was used. To obtain a better signal-to-noise ratio, an exponential window function for 0.3 Hz line broadening factor was applied to the free induction decays of NMR measurements. Chemical shifts are expressed as p.p.m. relative to an external standard of 3-(trimethylsilyl) propionic acid-d4.

### Crystallization of SPT

SPT was crystallized by the sitting drop vapor diffusion method in 24-well plates at 20 °C as described previously (22, 23). Briefly, an aliquot of 2 μl of 20 mg/ml protein solution was mixed with 4 μl of the reservoir solution containing 100 mM Tris–HCl (pH 8.5), 200 mM sodium acetate, and 19 to 24% (w/v) PEG4000. The drop was equilibrated against 500 μl of the reservoir solution for 2 days, and, then, the microbridge was transferred to a new 24-well plate, where the fresh reservoir solution contained 100 mM Tris–HCl (pH 8.5), 200 mM sodium acetate, and 13 to 18% (w/v) PEG4000. Plate-shaped or cube-shaped crystals were reproducibly grown within 2 to 5 days. We attempted to prepare the crystals of the binary complex with d-serine by soaking into the solution containing 100 mM Tris (pH 8.5), 200 mM sodium acetate, 15% (w/v) PEG4000, and 190 mM d-serine for 10 min. The crystals of the SPT–α-methyl-d-serine binary complex were prepared by two-step soaking method as described previously (23): The ligand-free form SPT crystals, which were obtained by soaking the crystals grown in the Tris-buffered solution into the Tris-free solution (100 mM Tricine-NaOH (pH 8.5), 200 mM sodium acetate, and 15% (w/v) PEG4000) for 90 min, were soaked again into the Tris-free solution containing 185 mM α-methyl-d-serine for 40 min before data collection.

### Data collection and structural determination

Crystals were cryo-protected by quick transfer through the soaking solution supplemented with 20% (v/v) ethylene glycol, then flash-frozen in liquid nitrogen or under a N_2_ gas cryostream (100 K). X-ray diffraction data were collected at the BL17A beamline at KEK Photon Factory, λ = 0.98 Å, and the BL38B1 beamline at SPring-8, λ = 0.9 Å.

All data were processed and scaled using XDS (60). Initial phases for each structure were determined by the molecular replacement method using MolRep software (https://www.ccp4.ac.ul/html/molrep.html) (61) in the CCP4 program suite (62) using PDB entry 3A2B as the search model after the removal of all water molecules. The model was refined using REFMAC5 (63) in the CCP4 suite, and manual adjustment and rebuilding of the model were performed using the program Coot (64). The quality of the structure was determined by MolProbity (65). Refinement statistics are summarized in Table 2. The atomic coordinates and crystal structures reported in the present study have been deposited in the Protein Data Bank with accession codes 8IYP and 8IYT.

### Structural analysis and generation of figures

Illustrations of the structures and sequence-independent structural superposition were performed using the PyMOL molecular graphics system (DeLano Scientific; https://pymol.org/2/).

## Data availability

All data are contained in the article and supporting information.

## Supporting information

This article contains Supporting information.

## Conflict of interest

The authors declare that they have no conflicts of interest with the contents of this article.
