# Supplementary material for: Racemization of the substrate and product by serine palmitoyltransferase from Sphingobacterium multivorum yields two enantiomers of the product from d-serine
Source: J Biol Chem. 2024 Feb 5;300(3):105728. doi: 10.1016/j.jbc.2024.105728 (PMC10912632; doi:10.1016/j.jbc.2024.105728)
Supplement: Supporting information [file mmc1.pdf]

Supporting Information

Racemization of the substrate and product by a bacterial serine palmitoyltransferase yields two enantiomers of the product from D-serine

Hiroko Ikushiro[1]*, Takumi Honda[2], Yuta Murai[2,3,4]*, Taiki Murakami[5], Aya Takahashi[5], Taiki Sawai[1], Haruna Goto[1], Shin-ichi Ikushiro[6], Ikuko Miyahara[5], Emi Ito[7], Yoshio Hirabayashi[8,9], Nobuo Kamiya[10], Kenji Monde[2,3]* and Takato Yano[1]*

[1] Department of Biochemistry, Faculty of Medicine, Osaka Medical and Pharmaceutical University, 2-7 Daigaku-machi, Takatsuki, Osaka 569-8686, Japan
[2] Graduate School of Life Science, Hokkaido University, Kita 21, Nishi 11, Kita-ku, Sapporo 001-0021, Japan
[3] Frontier Research Center for Advanced Material and Life Science, Faculty of Advanced Life Science, Hokkaido University, Kita21 Nishi11, Sapporo, Hokkaido 001-0021, Japan
[4] Division of Applied Bioscience, Graduate School of Agriculture, Hokkaido University, Kita9 Nishi9, Kita-ku, Sapporo, Hokkaido 060-8589, Japan
[5] Department of Chemistry, Graduate School of Science, Osaka Metropolitan University, 3-3-138 Sugimoto, Sumiyoshi-ku, Osaka, Osaka 558-8585, Japan
[6] Department of Biotechnology, Faculty of Engineering, Toyama Prefectural University, 5180 Kurokawa, Imizu, Toyama 939-0398, Japan
[7] RIKEN Cluster for Pioneering Research, RIKEN, Wako, Saitama 351-0198, Japan
[8] Institute for Environmental and Gender-Specific Medicine, Juntendo University Graduate School of Medicine, Chiba 279-0021, Japan
[9] RIKEN Cluster for Pioneering Research, RIKEN, Wako, Saitama 351-0198, Japan
[10] Research Center for Artificial Photosynthesis, Osaka Metropolitan University, 3-3-138 Sugimoto, Sumiyoshi-ku, Osaka, Osaka 558-8585, Japan

LIST OF SUPPORTING INFORMATION MATERIAL

Figure S1. Spectroscopic titration of the *S. multivorum* SPT with α-methyl-D-serine.

Supplementary Methods

Reference

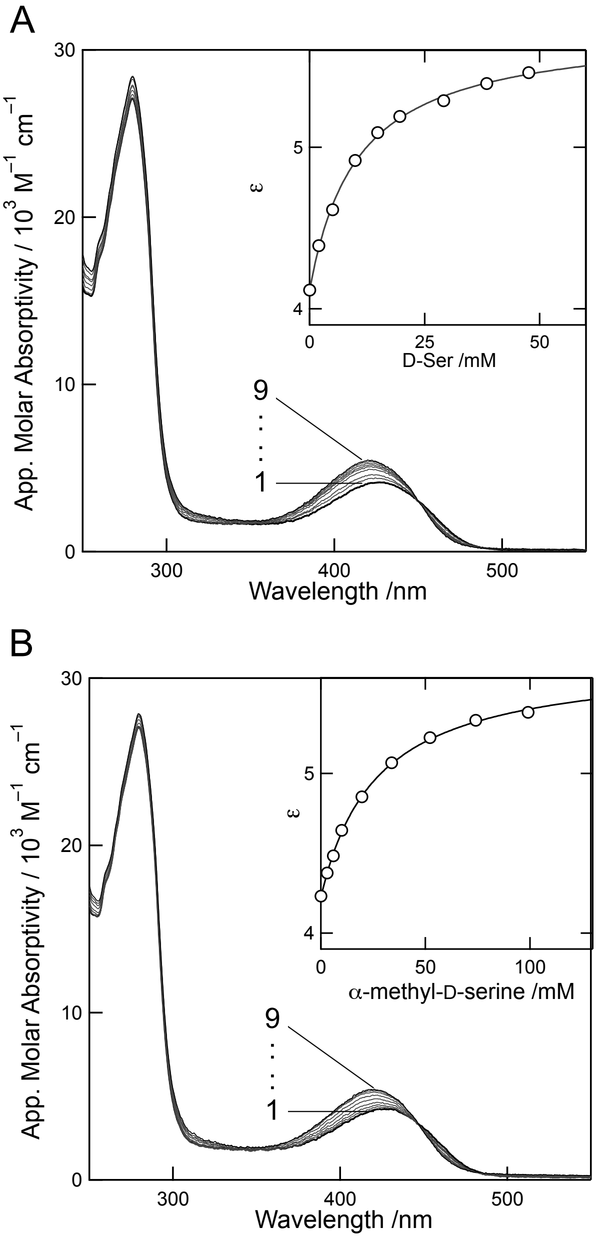

Figure S1. Spectroscopic titration of the *S. multivorum* SPT with D-serine or α-methyl-D-serine. (A) Absorption spectra of SPT (10 μM) in the presence of 0, 2.0, 5.0, 9.9, 15, 20, 29, 38, and 48 mM D-serine (lines 1–9, respectively). The inset shows the titration of SPT with α-methyl-D-serine monitored at 422 nm, fitted with a theoretical curve with a K_d value of 27 mM. (B) Absorption spectra of SPT (10 μM) in the presence of 0, 3.0, 6.0, 9.9, 20, 34, 52, 74, and 99 mM α-methyl-D-serine (lines 1–9, respectively). The inset shows the titration of SPT with α-methyl-D-serine monitored at 422 nm, fitted with a theoretical curve with a K_d value of 27 mM.

Supplementary Methods

Determination of dissociation constants (K_d) for amino acids.

The titration assay was carried out as previously reported (1). The changes of the 422 nm-absorption intensities of SPT upon addition of amino acids were plotted against the final concentrations of the amino acids, and the K_d values were calculated by fitting to a hyperbolic saturation curve using Igor Pro 6.37. software (Wave Matrix Inc., Lake Oswego, Oregon, United States). UV/Vis spectra of SPT were recorded with a Hitachi U-3310 spectrophotometer (Tokyo, Japan).

Synthesis of coumarin attached L- and D- 3-ketodihydrosphingosines (KDS)

***tert*-butyl (*S*)-2,2-dimethyl-4-palmitoyloxazolidine-3-carboxylate (2a)**

To a solution of 1a (226 mg, 1.14 mmol) in MeOH (10 mL) was added Pd/C and stirred overnight at room temperature under hydrogen atmosphere. The resulting mixture was filtrated by celite, and the crude mixture was purified by silica gel column chromatography (*n*-hexane to *n*-hexane/EtOAc = 10:1) to give 2a (181 mg, 79%) as yellow oil.

^{1}H NMR (500 MHz, CHLOROFORM-D) δ ppm 6.91 - 7.03 (m, 1H), 6.30 (d, *J* = 15.88 Hz, 1H), 4.39 - 4.73 (m, 1H), 3.87 - 4.22 (m, 2H), 2.23 (d, *J* = 7.08 Hz, 2H), 1.71 (s, 2H), 1.65 (s, 1H), 1.43 - 1.58 (m, 9H), 1.37 (s, 6H), 1.21 -1.31 (m, 20H), 0.88 (t, *J*= 6.84 Hz, 3H). The signal of primary hydroxy group was not observed. ^{13}C NMR (101 MHz, CHLOROFORM-D) δ ppm 208.68, 208.31, 152.43, 151.48, 95.17, 94.47, 80.92, 80.56, 65.75, 65.41, 65.35, 65.22, 39.10, 38.49, 31.97, 29.74, 29.72, 29.71, 29.70, 29.68, 29.64, 29.50, 29.46, 29.40, 29.32, 29.21, 28.38, 28.35, 26.23, 25.40, 24.88, 23.73, 23.18, 23.10, 22.71, 14.14 (Peaks in ^{13}C-NMR spectrum broad and split due to the presence of *N*-Boc rotamers). HRMS (*m/z*): $[M+Na]^+$ calculated for $C_{26}H_{49}NO_4Na$: 462.3553, found 462.3541.

tert-butyl (*R*)-2,2-dimethyl-4-palmitoyloxazolidine-3-carboxylate **(2b)** was obtained with the same manner (86%).

(*S*)-2-amino-1-hydroxyoctadecan-3-one·TFA salt (3a)

Compound 2a (75 mg, 0.17 mmol) and trifluoroacetic acid (500 µL) was stirred 2 h at 0 °C. Then, MeOH (3 mL) was added to the solution, and the reaction was stirred overnight at room

temperature. The solvent was evaporated and dried in vacuo to give 3a (63.9mg, 91%) as a white solid without further purification.

^{1}H NMR (500 MHz, METHANOL-D4) δ ppm 4.15- 4.18 (m, 1H), 3.93 - 4.13 (m, 2H), 2.64 (m, 2H), 1.63 (t, *J* = 7.09 Hz, 2H), 1.33 (brs, 24H), 0.91 (t, *J* = 6.84 Hz, 3H). ^{13}C NMR (101 MHz, METHANOL-D4) δ ppm 203.89, 60.85, 58.93, 38.29, 31.73, 29.46, 29.44, 29.43, 29.42, 29.40, 29.38, 29.27, 29.19, 29.13, 28.77, 22.85, 22.39, 13.09. HRMS (*m/z*): $[M+H]^+$ calculated for $C_{18}H_{38}NO_2$: 300.2897, found 300.2902. $[\alpha]_D$ +31.0 (*c* 1.0: methanol).

(*R*)-2-amino-1-hydroxyoctadecan-3-one·TFA salt **(3b)** was obtained with the same manner (95%). $[\alpha]_D$ –30.5 (*c* 1.0: methanol).

N-((S)-1-hydroxy-3-oxooctadecan-2-yl)-6-methoxy-3-oxo-3,8a-dihydro-2H-chromene-2-carboxamide (4a)

Compound 3a (20.8 mg, 52.5 μmol), DIPEA (11.0 μL, 57.7 μmol) and *N*-succinimidyl 7-methoxycoumarin-3-carboxylate (16.6 mg, 52.5. μmol) was dissolved in $CHCl_3$ (5 mL), and

stirred 2 h at room temperature. The resulting mixture was evaporated, and the residue was washed with saturated $NaHCO_3$ and dried over with $MgSO_4$. The crude mixture was purified by silica column chromatography (*n*-hexane/EtOAc = 1f:1) to give 4a (23.0 mg, 87%) as a white solid.

^{1}H NMR (400 MHz, CHLOROFORM-D) δ 9.63 (d, *J* = 6.9 Hz, 1H), 8.80 (s, 1H), 7.56 (d, *J* = 8.6 Hz, 1H), 6.93 (dd, *J* = 8.6, 2.2 Hz, 1H), 6.86 (d, *J* = 2.2 Hz, 1H), 4.77 (m, 1H), 4.03 (dd, *J* = 4.1, 1.7 Hz, 2H), 3.91 (s, 3H), 2.60 (m, 2H), 1.61 (t, *J* = 7.4 Hz, 2H), 1.22 (brs, 24H), 0.86 (s, 3H). The signal of primary hydroxy group was not observed. ^{13}C NMR (101 MHz, CHLOROFORM-D) δ 207.24, 165.25, 162.89, 161.55, 157.03, 148.83, 131.13, 114.31, 114.26, 112.34, 100.44, 63.23, 61.57, 56.16, 40.21, 32.01, 29.78, 29.77, 29.76, 29.74, 29.69, 29.54, 29.49, 29.44, 29.23, 23.53, 22.78, 14.21. HRMS (*m/z*): $[M+Na]^+$ calculated for $C_{29}H_{43}NO_6Na$: 524.2982, found 524.2969. $[\alpha]_D$ +13.5 (*c* 0.5: chloroform).

N-((*R*)-1-hydroxy-3-oxooctadecan-2-yl)-6-methoxy-3-oxo-3,8a-dihydro-2*H*-chromene-2-carboxamide **(4b)** was obtained with the same manner (83%). $[\alpha]_D$ –12.0 (*c* 0.5: chloroform).

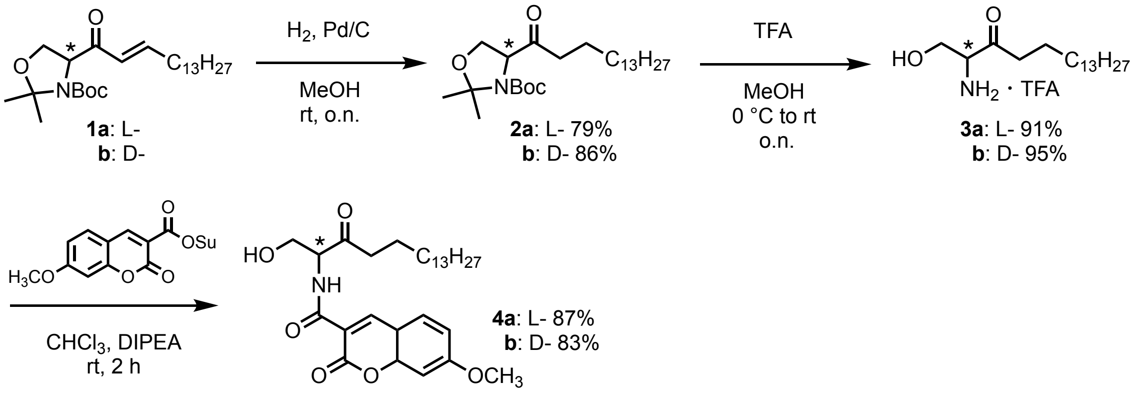

Scheme 1. Synthesis of coumarin attached L- and D-KDS

Reference

1. Ikushiro, H., Murakami, T., Takahashi, A., Katayama, A., Sawai, T., Goto, H., Koolath, S., Murai, Y., Monde, K., Miyahara, I., Kamiya, N., and Yano, T. (2023) Structural insights into the substrate recognition of serine palmitoyltransferase from *Sphingobacterium multivorum*. ***J Biol Chem*** **299**, 104684
